# Frontal alpha asymmetry: A potential biomarker of approach-withdrawal motivation towards pain

**DOI:** 10.3389/fpain.2022.962722

**Published:** 2022-09-27

**Authors:** Bárbara Silva-Passadouro, Ariane Delgado-Sanchez, James Henshaw, Karen Lopez-Diaz, Nelson J. Trujillo-Barreto, Anthony K. P. Jones, Manoj Sivan

**Affiliations:** ^1^ Academic Department of Rehabilitation Medicine, Division of Neuroscience and Experimental Psychology, University of Manchester, Manchester, United Kingdom; ^2^ Leeds Institute of Rheumatology and Musculoskeletal Medicine, University of Leeds, Leeds, United Kingdom

**Keywords:** pain catastrophising, EEG, biomarker, neurofeedback, placebo response

## Abstract

Pain-related catastrophising is a maladaptive coping strategy known to have a strong influence on clinical pain outcomes and treatment efficacy. Notwithstanding, little is known about its neurophysiological correlates. There is evidence to suggest catastrophising is associated with resting-state EEG frontal alpha asymmetry (FAA) patterns reflective of greater relative right frontal activity, which is known to be linked to withdrawal motivation and avoidance of aversive stimuli. The present study aims to investigate whether such a relationship occurs in the situational context of experimental pain. A placebo intervention was also included to evaluate effects of a potential pain-relieving intervention on FAA. 35 participants, including both chronic pain patients and healthy subjects, completed the Pain Catastrophising Scale (PCS) questionnaire followed by EEG recordings during cold pressor test (CPT)-induced tonic pain with or without prior application of placebo cream. There was a negative correlation between FAA and PCS-subscale helplessness scores, but not rumination or magnification, during the pre-placebo CPT condition. Moreover, FAA scores were shown to increase significantly in response to pain, indicative of greater relative left frontal activity that relates to approach-oriented behaviours. Placebo treatment elicited a decrease in FAA in low helplessness scorers, but no significant effects in individuals scoring above the mean on PCS-helplessness. These findings suggest that, during painful events, FAA may reflect the motivational drive to obtain reward of pain relief, which may be diminished in individuals who are prone to feel helpless about their pain. This study provides valuable insights into biomarkers of pain-related catastrophising and prospects of identifying promising targets of brain-based therapies for chronic pain management.

## Introduction

Pain-related catastrophising is a maladaptive cognitive-affective response to pain experiences characterized by the tendency to place emphasis on negative aspects of pain (1–3). Ample evidence points to the predictive value of this psychological construct on chronic pain severity and strong associations with poorer individual response to pain treatment (4–7). Higher pre-intervention catastrophising has been associated with potentiation of placebo-induced analgesic effects. Sullivan et al. (8) showed that higher tendency to catastrophise pain was associated with enhanced placebo analgesia response in neuropathic pain patients assigned to a placebo condition. In the active treatment group, higher catastrophising was associated with reduced response to treatment. This suggests catastrophising may have a differential impact on the outcomes of placebo intervention as opposed to active treatment.

With mounting evidence to support the significant effects of catastrophising on pain-related outcomes, researchers are drawing a new focus on the therapeutic applicability of catastrophising-targeted approaches. Psychological therapies known to reduce maladaptive catastrophic beliefs, such as cognitive-behavioural therapy, have shown great promise for managing pain (9–11). Notwithstanding the promising results, treatment effects are generally small and heterogenous across chronic pain conditions. An alternative approach would be to directly target underlying biological mechanisms *via* neuromodulation techniques, which offers the promise of non-invasive tailored interventions for chronic pain. The design of targeted brain-based therapies requires a deep understanding of the neural markers supporting catastrophic cognitions. One candidate neurophysiological signature is frontal alpha asymmetry (FAA), an indicator of lateralization of prefrontal cortex activity that measures the relative difference in alpha power between right and left frontal cortical regions (12). Alpha-band electroencephalography (EEG) oscillatory activity reflects functional inhibition of cortical processing (13). Therefore, greater FAA scores, for instance, indicative of lower relative left frontal alpha power are reflective of greater cortical activity over the left frontal hemisphere.

FAA is thought to reflect activation of two neurophysiological systems, the behavioural activation system (BAS) and the behavioural inhibition system (BIS), involved in coordinating distinct behavioural, emotional and cognitive responses to cues signalling either potential reward or punishment (12). This is dependent on the affective and motivational value the individual places on the stimulus. Activation of the two systems is reflected by asymmetric activity over the frontal brain regions and measured as FAA in EEG. Greater relative left frontal activity (indexed by greater FAA scores) is associated with enhanced activity of the BAS. BAS responds to signals of reward (14) and comprises brain regions that are involved in enhancing approach motivation or directing of one's behaviour toward positive desired stimuli (15). Right lateralized frontal activity (indexed by lower FAA scores), on the contrary, relates to engagement of the BIS. BIS responds to cues of potential punishment, such as pain, and involves brain regions associated with promoting withdrawal motivation or tendency to avoid or move away from aversive undesired stimuli (15). Considering catastrophising is regarded as a BIS-related pain-coping strategy, a link between this psychological construct and greater relative right frontal activity seems logical according to the approach-withdrawal motivational model of FAA (also referred to as BIS-BAS model). This has been shown in a prospective study of spinal cord injury patients where greater resting-state relative right frontal activity was associated with trait catastrophising measured two years after EEG recordings (16).

The present study aims to test the relationship between FAA elicited during an experimental pain paradigm and individual trait catastrophising. In more detail, this study investigates how relative activation of the BIS or BAS in a situation known to induce pain (as reflected by EEG FAA scores) relates to an individual's tendency to catastrophise their pain and engage with this withdrawal-associated coping strategy. This study is a secondary analysis of data collected for the “The Effectiveness of Neurofeedback for the Treatment of Chronic Pain” study (pre-registration reference: NCT04097522).

Placebo was used to study the effects of a potential pain-relieving intervention on FAA. EEG was recorded whilst participants performed tonic cold pressor tests (CPT) with or without prior application of placebo analgesic cream. FAA was measured as the relative difference between EEG alpha power in the right frontal region and EEG alpha power in the left frontal region. It was hypothesized that: 1. Individual tendency to catastrophise pain correlates negatively with FAA. This meaning that greater catastrophic tendency associates with lower FAA, reflective of greater relative right frontal activity; 2. FAA scores decrease during pain, relative to resting-state. This is expected to occur as a reflection of activation of the BIS (indexed by greater relative right frontal activity) and an increased urge to escape or withdrawal motivation in response to the painful aversive stimulus; 3. Placebo cream application induces greater changes in FAA scores in individuals with higher catastrophic tendency, as they are expected to demonstrate stronger placebo response. To the extent that FAA can be successfully manipulated by neurofeedback-training techniques (17, 18), advances in assessing the validity of FAA as a neurophysiological correlate of pain-related catastrophising may reveal novel promising targets of non-invasive therapeutic strategies for the management of chronic pain.

## Materials and methods

### Participants

The original study involved 35 volunteers: 28 patients with diagnosis of chronic pain conditions (22 females; age, 59.9 ± 14.55; mean ± SD) and 7 unmatched healthy subjects (6 females; age, 56.8 ± 14.97; mean ± SD). Patients presented with back and/or hip pain (*N* = 12), fibromyalgia (*N* = 9), arthritis (*N* = 6) and other less prevalent chronic pain conditions (e.g. migraine); with 29% (*N* = 8) of patients presenting two or more comorbid pain conditions.

The criteria for selection included: [1] clinically significant pain persisting longer than 3 months, for patients; and [2] no clinical history of pain, for healthy subjects. Subjects involved in clinical trials; with current or scheduled hospitalization; with history of brain injury, stroke, neurological procedure or implanted neurostimulator; with high or uncontrolled blood pressure; non-English speaking or with damaged skin on the head were excluded from participation. Selected participants were required to provide informed consent prior to the experiments and were informed of their right to withdraw from the study at any time. All procedures in the original study were conducted in accordance with the Declaration of Helsinki. The study was registered with clinicaltrials.gov (pre-registration reference: NCT04097522) and approved by The Health Research Authority (IRAS ID: 244779) and The North of Scotland Research Ethics Committee 2 (18/NS/0102).

### Questionnaires

The subjects were asked to fill out the self-assessment questionnaires listed below (prior to the experiments):

(a) Pain Catastrophising Scale (PCS) – measures the level of catastrophising by asking subjects to rate the frequency to which they experienced certain thoughts and feelings around pain during past painful experiences (2). It has been widely used in research settings to reliably assess the level of catastrophic thinking around pain in clinical and non-clinical populations (19–21). The self-report PCS questionnaire comprises 13 statements relevant to the three dimensions of catastrophising: Rumination, which refers to the attentional bias towards pain and inability to suppress negative thoughts around it (e.g. “I keep thinking about how badly I want the pain to stop.”); Magnification, which refers to exaggeration of pain-related sensations (e.g. “I wonder whether something serious may happen.”); and Helplessness, which refers to feelings of inability to cope with pain (e.g. “There”s nothing I can do to reduce the intensity of the pain.”). The 3-component model of the PCS is supported by confirmatory factor analysis (20–22). Ratings are provided on a scale of 0 (=not at all) to 4 (=all the time). Higher PCS scores (range from 0 to 52) reflect a greater degree of pain-related catastrophising. The total scores were separated into rumination, magnification and helplessness subscales for analysis.
(b) Hospital Anxiety and Depression Scale (HADS) – detects and evaluates severity of anxiety and depression in clinical settings (23–25). It consists of 14 items, scored on a 4-point response scale, concerning emotional states associated with the two common psychological disturbances. The HADS scores were separated into Anxiety and Depression for analysis (each ranging from 0 to 21).

### Experimental tonic cold pain

#### Cold pressor test

Tonic pain was experimentally induced using the cold pressor test paradigm. Participants were asked to place the left hand in a container filled with crushed ice at 5 or 10 °C and maintain it for up to 3 min. They were informed they could withdraw the hand from the ice bath at any point during the CPT condition if the pain became too unbearable. During hand submersion, subjective pain ratings were obtained at 30 s intervals using the Visual Analogue Scale (VAS). The VAS measures self-reported pain intensity on a scale ranging from 0 (=no pain) to 10 (=unbearable pain) (26). In order to prevent introduction of speech artifacts in the EEG recording, participants were instructed to point with their right hand at the mark corresponding to the perceived pain intensity on a printed copy of a VAS.

#### Experimental procedure

**Figure 1:**
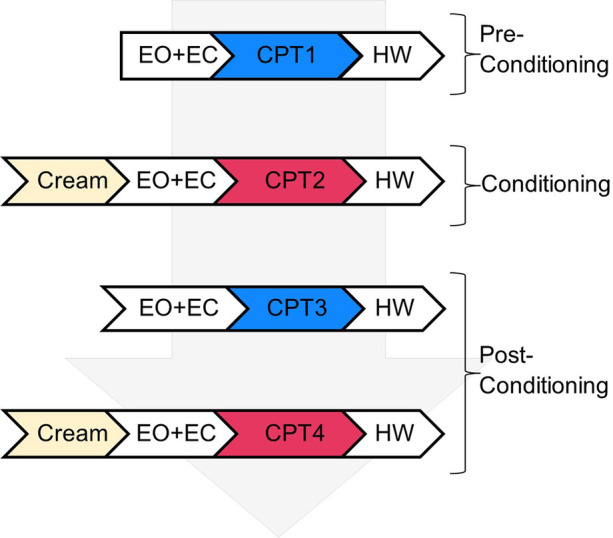
Experimental design chart outlining the steps in a single experimental block comprising four cold-pressor test (CPT) submersions. Each block consisted of three stages: pre-conditioning, with an initial CPT submersion (CPT1); conditioning, induced by application of placebo cream prior to a second CPT submersion (CPT2 where temperature was raised surreptitiously by 5 °C); and post-conditioning, with two final CPT conditions (CPT3 and CPT4 without and with prior application of placebo cream, respectively). Note that each CPT submersion was preceded by resting state eyes open (EO) and eyes closed (EO) conditions for baseline EEG recordings and followed by submersion of the hand in warm water - hand warming (HW) time.

Participants attended a single laboratory session that consisted of a total of 8 CPT submersions separated into two blocks: one with ice bath at 5 °C and another at 10 °C, carried out in randomized order across subjects. Throughout the experimental procedure, participants were sat comfortably with the EEG cap fitted to their head. Prior to each CPT submersion, two minutes each of eyes open (EO) and eyes closed (EC) EEG baseline data were recorded. Resting state, as mentioned throughout the paper, refers to the baseline eyes open condition just before the first CPT, prior to any experimental tonic pain or placebo manipulation. Each block consisted of four CPT conditions in the following order (Figure 1): (1) experimental induction of tonic cold pain prior to placebo intervention (CPT1), (2) hand submersion following application of inert treatment with surreptitious manipulation of ice bath temperature (CPT2), (3) hand submersion without prior application of placebo cream (CPT3), and (4) novel application of the inert cream without surreptitious temperature manipulation to test placebo effects (CPT4). Continuous EEG was recorded while subjects immersed the hand in the ice bath in each CPT condition. Participants were given 5 min to warm up the hand in a bucket with hot water at 40 °C after each CPT submersion (hand warming (HW) time).

#### Placebo intervention

An inert aqueous cream was used to manipulate the participant's expectations on the experience of pain during CPT that may lead to alterations in their response to and perception of pain. The inert cream had no analgesic or any other therapeutic properties and, therefore, no expected secondary effects on the physiological response to immersion of the hand in iced water. Participants applied the placebo cream to their left hand and wrist prior to submersion of the hand in the second and fourth CPT conditions of each block (CPT2 and CPT4, respectively). They were informed they would be testing the effectiveness of a cream that “may or may not contain a local anaesthetic”. The conditioning procedure consisted of surreptitious temperature manipulation in CPT2 only (where ice bath temperature was raised by 5 °C without the participants' knowledge) to create impression of pain relief and further induce belief of analgesic efficacy. Placebo response was calculated as the difference between mean pain ratings obtained during initial CPT and mean ratings regarding placebo CPT.

### EEG recordings

EEG activity was recorded using a 64-channel BrainAmp EEG system with Ag/AgCl electrodes, attached to a MR cap according to the standard 10–20 system (27), and DC/MR amplifiers. The recorded scalp sites were: Fp1, Fp2, F3, F4, C3, C4, P3, P4, O1, O2, F7, F8, T7, T8, P7, P8, Fz, Cz, Pz, Oz, FC1, FC2, CP1, CP2, FC5, FC6, CP5, CP6, TP9, TP10, POz, ECG, F1, F2, C1, C2, P1, P2, AF3, AF4, FC3, FC4, CP3, CP4, PO3, PO4, F5, F6, C5, C6, P5, P6, AF7, AF8, FT7, FT8, TP7, TP8, PO7, PO8, FT9, FT10, Fpz, CPz, FCz and AFz. The FCz electrode was used as reference, and the AFz electrode as ground reference. EEG recording was conducted at a sampling rate of 1000 Hz and impedances were kept below 20 kΩ.

#### EEG data processing and frequency analysis

Pre-processing and data cleaning was conducted using the EEGLAB software toolbox for MATLAB (ver. R2020b) (28). High- and low-pass filters were applied to raw EEG data (resulting in a 0.5 to 35 Hz frequency bandwidth) followed by removal of 50 Hz interfering frequency using notch filtering. The data were then re-referenced using the common average reference method (29). Flat or excessively noisy channels were removed from the whole session dataset *via* interpolation. Continuous data for each participant was separated into 1 s epochs. Removal of eye movement and blink artifacts was performed using Independent Component Analysis implemented in EEGLAB plugin SASICA (30). The final step consisted of visual inspection and manual rejection of any remaining trials containing strong artifacts.

Frequency analysis of the pre-processed data was carried out by means of an in-house MATLAB code which used the Fieldtrip toolbox functionality (31). For the purpose of the present study, the Fast Fourier Transform (FFT) algorithm was used to calculate the spectral power in the alpha frequency band (8–12 Hz) for all EEG epochs and all channels at resting state and during CPT submersions. Hanning window smoothing (0.5 Hz) was applied to reduce spectral leakage effects (32). The alpha power of EEG electrodes F3 (left hemisphere) and F4 (right hemisphere) was log-transformed (20*Log_10_[absolute power]) (33) and the FAA score was calculated by subtracting the value at F3 from the value at F4 (20*Log_10_[F4] − 20*Log_10_[F3]) (12). Averaging of FAA scores across segments was performed to obtain a single FAA score for each participant for each of the conditions under analysis. To test the regional specificity of the FAA score, parietal alpha asymmetry (PAA) scores were computed based on electrodes P3 and P4 (20*Log_10_[P4] − 20*Log_10_[P3]) following the protocol described above for FAA.

### Statistical analysis

Data analysis focused on resting state EEG recordings and the first two CPT conditions of each block (CPT1 and CPT2 referred to as “initial CPT” and “placebo CPT”, respectively) of the original experimental procedure. Statistical analysis was carried out using SPSS (version 25). Shapiro-Wilk tests were performed prior to computation of other statistical tests to assess if data followed a normal distribution. When data was found to be non-normally distributed, non-parametric tests were applied. The significance level for all statistical tests was set to *p* < 0.05. Removal of the hand from the ice bath prior to completion of the 3 min of CPT resulted in the individual participant's data being excluded from that specific CPT condition. On the same note, participants that did not complete all questionnaire items were excluded from analysis involving that specific psychometric variable. Correlations between psychological variables were measured using the Spearman's rank-order correlations coefficient.

The primary aim of statistical analysis was to investigate the relationship between FAA and PCS scores reported prior to the experimental procedure in the situational context of experimental tonic cold pain. Spearman's rank-order correlation tests (two-tailed) were conducted between each subscale of the PCS questionnaire and FAA scores relevant to resting state and initial CPT conditions for each block (5 °C and 10 °C). The same statistical test was used for PAA to assess the regional specificity of results. Due to significant correlations between psychometric variables, the independence of the relationship between FAA and PCS scores was tested by computing a non-parametric Spearman's partial correlation test corrected for the effects of HADS-Anxiety and Depression scores simultaneously. The null hypothesis of no difference in mean FAA scores between resting state and the initial CPT condition was also tested by means of a Paired-samples t test (two-tailed), computed for each block separately. To assess the effects of temperature, the change in FAA scores elicited by experimental tonic cold pain was compared between the 5 °C and the 10 °C Blocks using a Paired-samples t test (two-tailed).

To determine whether placebo intervention has an impact on FAA and its relationship with PCS scores, correlational analysis (Spearman's rank correlation) was performed between FAA scores relevant to the placebo CPT condition and each subscale of the PCS questionnaire. The effects of placebo treatment on FAA were assessed by testing the null hypothesis of no difference in mean FAA between the initial and the placebo CPT conditions using a Paired-samples t test (two-tailed). Where participants' data was grouped based on PCS-subscale scores, mean values were used to differentiate high vs. low scorers, with low scorers reporting PCS levels below the mean and high scorers above the mean.

Data and study materials including the study analysis code are available on request by contacting the corresponding author.

## Results

### Psychometric variables and pain ratings

Twenty-seven participants completed the PCS questionnaire with total scores, in the range from 0 to 52, averaging 14.13 ± 2.72. Mean values (±SD) for each PCS subscale were as follows: 4.89 ± 4.69 for PCS-rumination; 2.07 ± 2.96 for PCS-magnification; and 5.70 ± 6.26 for PCS-helplessness. HADS-Depression scores correlated positively with PCS subscales rumination [*r_s_*(*N* = 24) = 0.451, *p* = 0.027] and helplessness [*r_s_*(*N* = 24) = 0.498, *p* = 0.013] only. No evidence was found for significant correlations between HADS-Anxiety scores and any of the three PCS subscales. There was a statistically significant association between HADS-Anxiety and Depression scores [*r_s_*(*N* = 27) = 0.395, *p* = 0.041].

With respect to behavioural outcomes, average pain ratings across participants during the initial CPT were 5.67 ± 2.29 and 4.07 ± 2.13 with ice bath at 5 °C and 10 °C, respectively. In the placebo CPT, participants rated pain intensity on average 3.88 ± 2.61 in the 5 °C Block and 2.21 ± 1.80 in the 10 °C Block, with a mean placebo response of −0.43 ± 0.84 and −0.21±-0.82, respectively.

### Association between interindividual variability in FAA and PCS subscale scores

**Figure 2:**
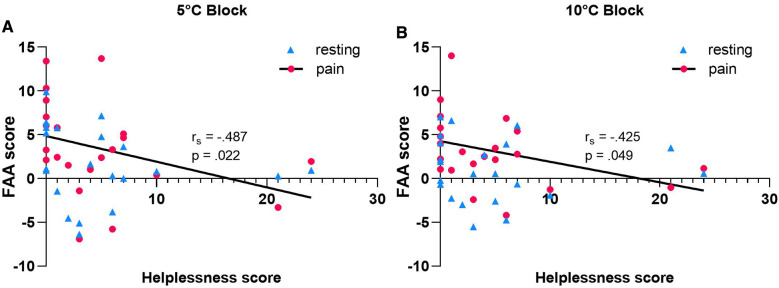
Relationship between pre-intervention PCS-helplessness scores and FAA recorded at resting state eyes open condition vs. tonic cold pain at 5 °C **(A)** or 10 °C **(B)**. PCS-helplessness is significantly negatively correlated with FAA scores relevant to initial CPT condition in both 5 and 10 °C Blocks (*N*- = 22). Note that no statistically significant correlation was found at resting state.

Multiple Spearman's rank-order correlations were carried out to assess whether interindividual variability in FAA was associated with PCS scores reported prior to the experiment. No statistically significant results were found with respect to the PCS subscales rumination and magnification in the 5 or 10 °C CPT conditions [rumination: *r_s_*(*N* = 22) = −0.343, *p* = 0.118 for the 5 °C Block; *r_s_*(*N* = 22) = −0.351, *p* = 0.109, for 10 °C Block] [magnification: *r_s_* (*N* = 22) = −0.157, *p* = 0.485 for the 5 °C Block; *r_s_*(*N* = 22) = −0.333, *p* = 0.130 for the 10 °C Block]. During tonic cold pain, individual FAA was associated exclusively with the PCS subscale helplessness. There was a strong negative correlation between the two factors regardless of temperature of the ice bath, with lower FAA associating with higher helplessness scores [5 °C Block: *r_s_*(*N* = 22) = −0.487, *p* = 0.022; 10 °C Block: *r_s_*(*N* = 22) = −0.425, *p* = 0.049] (Figure 2). When controlled for HADS-Anxiety and Depression scores, the significant correlation persisted only for the 5 °C initial CPT condition [*r_s_* (*N* = 15) = −0.642, *p* = 0.005]. No statistically significant association was found between PCS-helplessness and PAA [*r_s_*(*N* = 22) = 0.176, *p* = 0.432], suggesting that the inverse relationship between PCS-helplessness and alpha asymmetry is specific to frontal regions.

**Figure 3:**
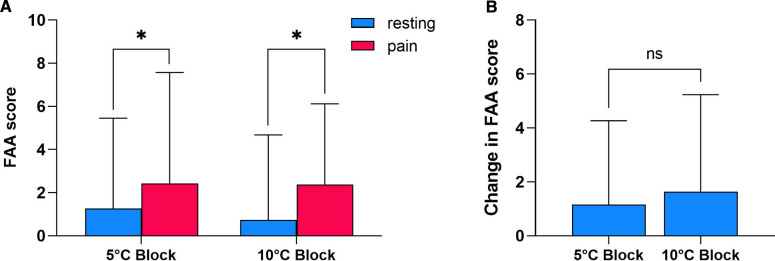
Effects of CPT-induced tonic pain on FAA. **(A)** Comparison between FAA scores recorded at resting state eyes open condition vs. during tonic cold pain induced by hand submersion in ice bath at 5 or 10 °C (*N* = 31). **(B)** Change in FAA scores (initial CPT condition – resting state) elicited by CPT at 5 °C vs. 10 °C (*N* = 31). Data shown as mean ± SD. * *p* < 0.05; ns- Not Significant.

Whilst meaningful during initial CPT conditions, the association between FAA and helplessness failed to reach statistical significance at resting state, which may be indicative of changes in FAA throughout the experimental procedure [5 °C Block: *r_s_*(*N* = 22) = −0.416, *p* = 0.054; 10 °C Block: *r_s_*(*N* = 22) = −0.184, *p* = 0.413]. To further evaluate the effects of CPT-induced pain on FAA, a Paired-samples t test was conducted between resting state FAA scores and FAA scores recorded during pain (Figure 3A). There was a statistically significant increase in mean FAA scores from baseline (1.266 ± 4.19) to pain state (2.430 ± 5.14) in the 5 °C Block [*t*(31) = 2.122, *p* = 0.042]. A significant increasing tendency was also observed following hand submersion in the 10 °C ice bath (resting-state FAA: 0.737 ± 3.95; initial CPT FAA: 2.383 ± 3.74) [*t*(31) = 2.593, *p* = 0.014]. The average change in FAA scores induced by CPT did not differ significantly between blocks (5 °C Block: 1.164 ± 3.10; 10 °C Block: 1.647 ± 3.59) [*t*(31) = −0.644, *p* = 0.525] (Figure 3B).

### Effects of placebo intervention on FAA and its association with helplessness

**Figure 4:**
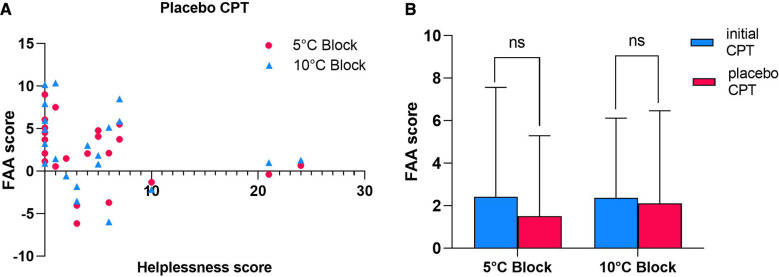
Effects of placebo intervention on FAA and its association with pre-intervention PCS-helplessness scores. **(A)** Relationship between pre-intervention PCS-helplessness scores and FAA recorded during tonic cold pain after application of placebo cream (placebo CPT) induced by CPT at 5 or 10 °C (*N* = 22). Note that no statistically significant correlation was found during placebo CPT conditions. **(B)** Comparison between FAA scores recorded during pre-placebo intervention CPT condition (initial CPT) vs. during CPT condition following application of placebo cream (placebo CPT) in 5 or 10 °C Block (*N* = 31). Data shown as mean ± SD. ns- Not Significant.

The second main purpose of the study was to examine how catastrophising modulates the effects of placebo intervention on FAA. Spearman's correlation analysis revealed that the statistically significant inverse relationship between FAA and PCS-subscale helplessness scores was lost when placebo cream was applied prior to the CPT [5 °C Block: *r_s_*(*N* = 22) = −0.382, *p* = 0.079; 10 °C Block: *r_s_*(*N* = 22) = −0.261, *p* = 0.240] (Figure 4A). Despite a decreasing tendency, no evidence was found for a significant reduction in mean FAA scores between the initial CPT (2.430 ± 5.14) and the placebo CPT (1.510 ± 3.78) in the 5 °C Block [*t*(31) = 1.733, *p* = 0.093] or the 10 °C Block [*t*(31) = 0.511, *p* = 0.613] (Figure 4B).

To further investigate the loss of the significant relationship between FAA and PCS-helplessness and why mean FAA scores do not differ significantly between the initial CPT and the placebo CPT, participants were separated according to individual pre-treatment PCS-helplessness scores with low helplessness scorers reporting PCS-helplessness scores below the mean (5.70). Data from the 10 °C Block was not included in further analysis as the relevant FAA scores did not appear to be independently associated with PCS-helplessness after removing the effects of anxiety and depression.

**Figure 5:**
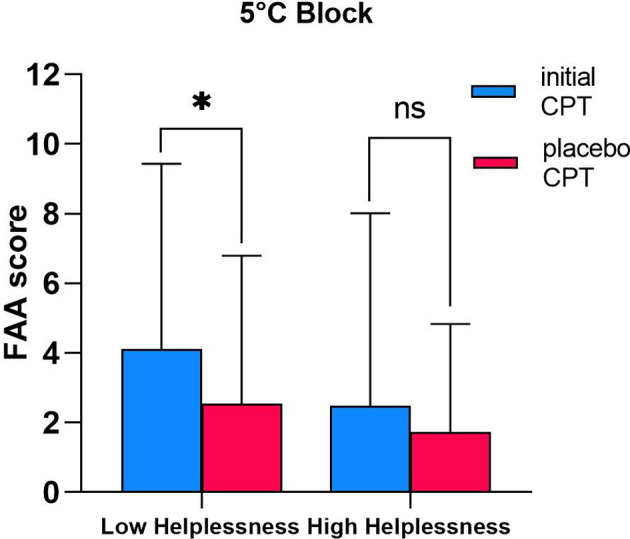
Differential effects of placebo treatment on FAA in low vs. high helplessness scorers. Comparison between FAA scores recorded during pre-placebo intervention CPT condition (initial CPT) vs. during CPT condition following application of placebo cream (placebo CPT) in individuals scoring low (*N* = 15; PCS-helplessness score <5.70) and high (*N* = 7; PCS-helplessness score >5.70) on PCS-subscale helplessness. Only data relevant to the 5 °C Block is presented in this graph. Data shown as mean ± SD. * *p* < 0.05; ns- Not Significant.

Strong evidence supporting differential effects of placebo intervention was found with low helplessness scorers (PCS-helplessness score <5.70, *N* = 15) showing a statistically significant decrease in FAA between the initial CPT (4.642 ± 5.54) and the placebo CPT condition (2.795 ± 3.99) [*t*(14) = 2.275, *p* = 0.039] (Figure 5). Whilst this was true for participants scoring below mean on PCS-subscale helplessness, no significant difference between the two conditions (initial CPT FAA: 0.903 ± 4.10; placebo CPT: 0.946 ± 3.12) was evident in high helplessness scorers (PCS-helplessness score >5.70, *N* = 7) [*t*(6) = −0.062, *p* = 0.953].

## Discussion

This study, to the best of our knowledge, provides the first comprehensive assessment of the relationship between PCS-assessed catastrophising subdomains and the electrophysiological marker FAA in the context of experimental pain and placebo. Consistent with Jensen et al. observations (16), this research demonstrates an association between FAA and tendency to experience pain-related catastrophising cognitions. The expected inverse relationship with FAA was only observed in regard to pre-procedure helplessness subscale of PCS and not the other two subscales of rumination and magnification. This study also demonstrated that FAA increases in response to experimentally-induced pain. When placebo cream was applied, FAA decreased in individuals who are less prone to catastrophic helplessness, but not in those with a higher tendency to experience feelings of helplessness.

Helplessness denotes beliefs of loss of control frequently leading to resignation to one's own pain, thus the approach-withdrawal motivation model for FAA (12) predicts the association between greater relative right frontal activity and helplessness. This is in keeping with the results of this study, which demonstrate that individuals who are prone to catastrophic helplessness cognitions tend to respond with greater relative right frontal cortical activity when exposed to pain (as indexed by lower FAA scores). These findings suggest that the helplessness element of catastrophising may be supported by enhanced assembly of neuronal activity in the right frontal cortex and consequent imbalance between the two neural motivational systems BIS and BAS (15). Within the framework of chronic pain, BIS hyperactivation may facilitate a maladaptive coping strategy that disposes the individual to react to ongoing pain *via* general inhibition of behaviour, decreased approach motivation and avoidance responses. If reiterated by future studies on chronic pain patient-only samples, these results may add to a growing body of evidence that support the BIS-BAS model for chronic pain proposed by Jensen et al. (34), that may be useful in explaining helplessness-related behaviours such as anhedonia and social withdrawal often experienced by chronic pain patients.

It should be noted that only individual differences in FAA during experimental tonic cold pain show a meaningful relationship with the psychological factor of helplessness. This is in accordance with the capability model advanced by Coan et al. (35) that states that individual differences in FAA are more pronounced when EEG is recorded in situational context as opposed to resting conditions, thus more powerfully reflecting predisposition towards a particular psychopathology (36). It can therefore be suggested that helplessness-relevant variance in FAA arises in situations assumed to induce helplessness cognitions.

Also consistent with the FAA motivational model is the negative direction of associations with the other PCS subcategories. However, failure to reach statistical significance suggests the rumination and magnification domains of pain-related catastrophising, while not excluding the possibility of some degree of overlap, may be associated with different electrophysiological mechanisms. These findings add to a growing consensus that the influence of tendency to catastrophise on particular pain-related outcomes may be more powerful when explained on the basis of the individual subdomains rather than the global multifactorial construct of catastrophising (37–39).

Another important finding was that FAA is modulated by experimental tonic cold pain. When interpreted from the perspective of absence vs. presence of stimuli, the observed increase in relative left frontal activity, as indexed by greater FAA scores, could be attributed to allocation of attention driven by approach motivation to an introduced emotionally salient stimulus (40). The incremental tendency did not appear to depend on pain intensity, which increases along with decreases in temperature of tonic cold pain stimulus, but rather on the experience of pain itself. Although the opposite direction of response was expected, these results do not necessarily negate the initial hypothesis. Previous studies have reported differential effects of negative, neutral and positive emotional stimuli on FAA (41, 42). It could therefore be argued that, when compared to emotionally-neutral or non-aversive stimuli, FAA decreases in response to the threat value of pain facilitating avoidance behaviours and urge to escape pain. Another possible explanation for a greater relative left frontal activity during experimental pain is the bias towards finding an internal solution to ease pain, prompted by the motivational drive to obtain reward of pain relief.

The proposed duality corroborates the ideas of Fields (43), who suggests the existence of conflicting motivations in pain. Pain induces both withdrawal and approach-motivated behaviours prompted by negative and positive affective states that respond to the aversiveness of pain or reward of pain relief, respectively. Furthermore, according to his motivation-decision model of pain, the value one places on the threat aspect of pain as opposed to predicted reward of relief will determine which neural motivational system is selected and, ultimately, which behaviour (approach or withdrawal) is facilitated. In this instance, individuals who are more prone to thoughts of helplessness may manifest motivational drive but to a lesser extent, as they place less value on the reward of pain relief, which further confirms the observed inverse relationship between FAA and helplessness. Trait or dispositional pain-related helplessness may therefore be regarded as an individual's ability or recurrent behavioural, cognitive or affective tendency for approach or withdrawal response to painful events.

This line of reasoning also provides a logical explanation for the outcomes of placebo intervention. When provided with a potential external solution for the pain, the motivational drive to obtain reward of pain relief is eased, as participants stop trying to find an internal solution, in association with a reduction in relative left frontal activity (indexed by lower FAA scores). Nevertheless, placebo intervention showed no statistically significant effects on FAA. An alternative conceptual framework was proposed to test the hypothesis that placebo intervention has differential effects on FAA depending on the individual's tendency to feel helpless about their pain. Given that no reports of cognitions and feelings of helplessness were taken throughout the experiment, the indirect effects of placebo intervention targeted at reducing pain intensity on the levels of state catastrophising-associated helplessness were not explored. Contrary to expectations concerning global catastrophising, higher propensity to catastrophic-associated helplessness cognitions did not predict a stronger FAA response to placebo intervention. In fact, application of placebo cream prior to hand submersion did not seem to produce significant effects in participants scoring high on PCS-helplessness. A possible explanation for this might be that high helplessness scorers assume a passive coping strategy towards pain, as postulated by Snow-Turek et al. (44). As less value is placed on the reward of pain relief, these individuals do not attempt to deal with their pain *via* internal processes, but instead rely on external resources and others, which could be interpreted as a diminished ability of self-regulation of pain. Therefore, introducing a placebo treatment will not ease attempts to self-control their own pain as they do not engage with this approach-oriented behaviour towards pain in the first place.

This study has few limitations. The surreptitious temperature manipulation may have introduced a confounding variable that contributes to the observed changes in FAA during the placebo CPT condition. Further analysis with more focus on the fourth CPT condition of each experimental block (post-conditioning hand submersion with prior application of placebo cream but no surreptitious increase of ice bath temperature) is therefore suggested in future studies, although the potential effects of learning should be accounted for when designing the research study. Another limitation to this study is the small sample size which restricted computation of statistical comparisons between groups based on participant status or PCS-helplessness scores. It is known that, as pain becomes chronic, structural and functional changes occur in cortical areas involved in processing the emotional and motivational aspects of pain that may lead to abnormal frontal interhemispheric activity (45). There is therefore a potential for bias from the unequal number of chronic pain patients and healthy subjects in the sample which may limit the generality of findings. Future studies with larger sample sizes may examine modulation of FAA during pain and relationship with catastrophising-associated helplessness based on participant status (i.e. chronic pain patients group and healthy subjects group analysed separately). The predominance of females in the sample may have also introduced an element of bias as few studies have shown that females tend to catastrophise more readily in pain situations than males (46, 47). The potential influence of gender differences in FAA should also be accounted for (48, 49). Finally, an issue that was not addressed in this study was whether FAA associated with subjective pain intensity in the initial CPT and changes in pain ratings following application of a placebo cream. Future studies on this question could shed more light on the potential of targeting FAA for the clinical management of pain.

## Conclusion

The research findings in the present study provide a new understanding of FAA biomarker as a reflection of the motivational drive to obtain reward of pain relief, that appears to be compromised in those who are prone to feel helpless about their pain. This has particular significance as it supports the relevance of psychological interventions aimed at reducing catastrophic helplessness cognitions, in particular CBT. Whilst further evidence is needed to confirm these findings, this paper provides insights for the development of promising non-invasive non-pharmacological therapeutic strategies for the management of chronic pain. Neurofeedback techniques that train individuals to self-modulate FAA towards a pattern reflective of greater relative left frontal activity may promote a more active coping approach towards pain in those who are prone to catastrophic helplessness. Further research is required to establish the therapeutic applicability of these neuromodulation strategies, either in combination or as an alternative to currently available pharmacological treatment, to improve pain-related outcomes in chronic pain populations.

## Data Availability

The raw data supporting the conclusions of this article will be made available by the authors, without undue reservation.
